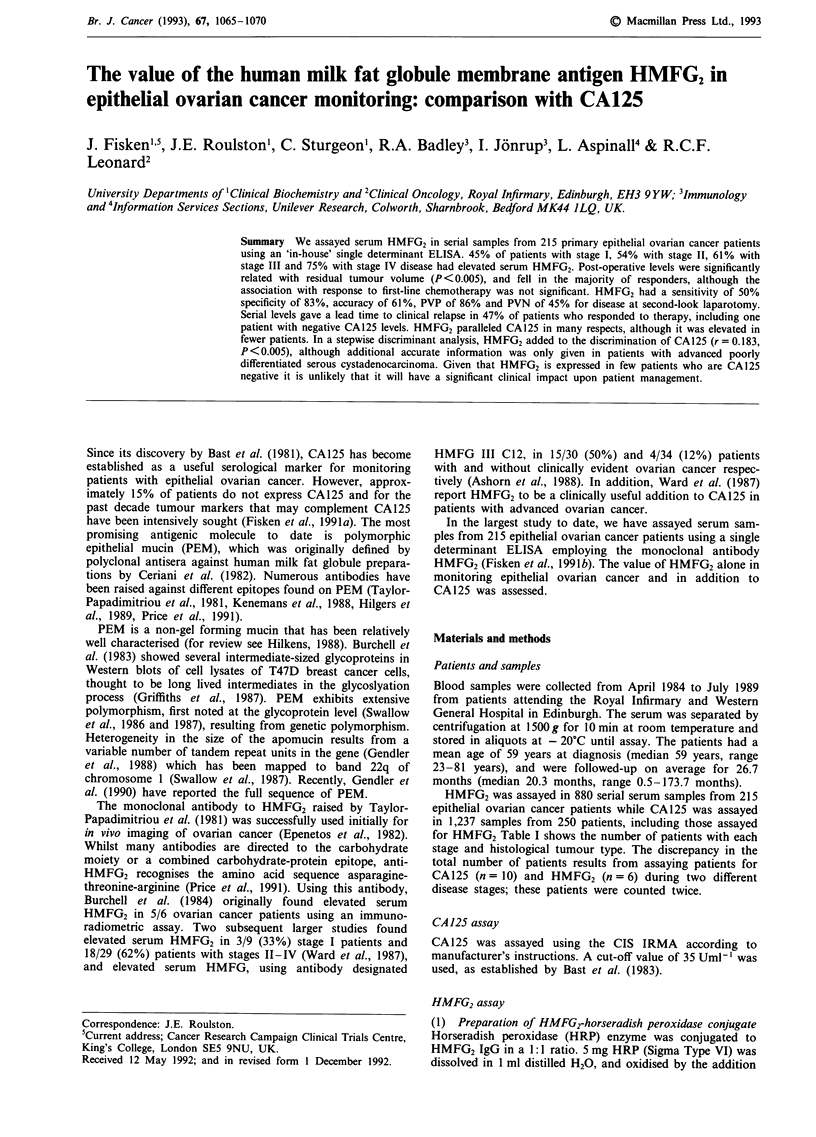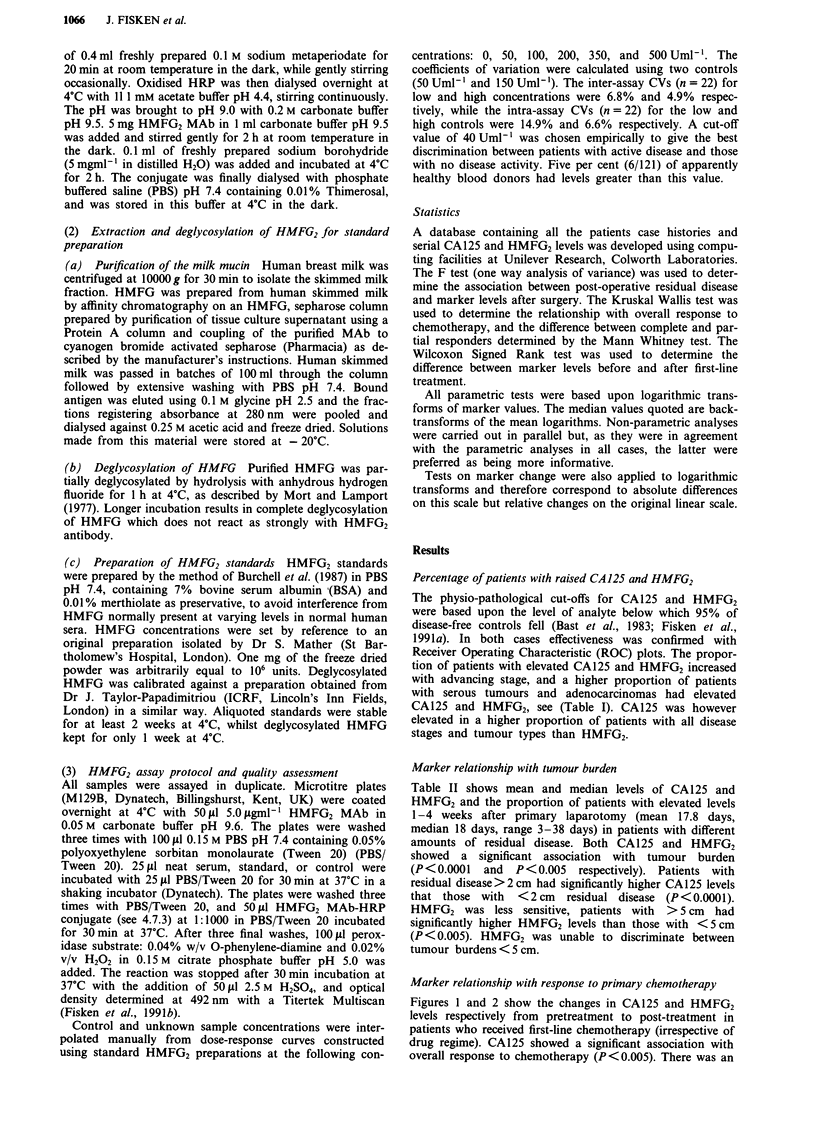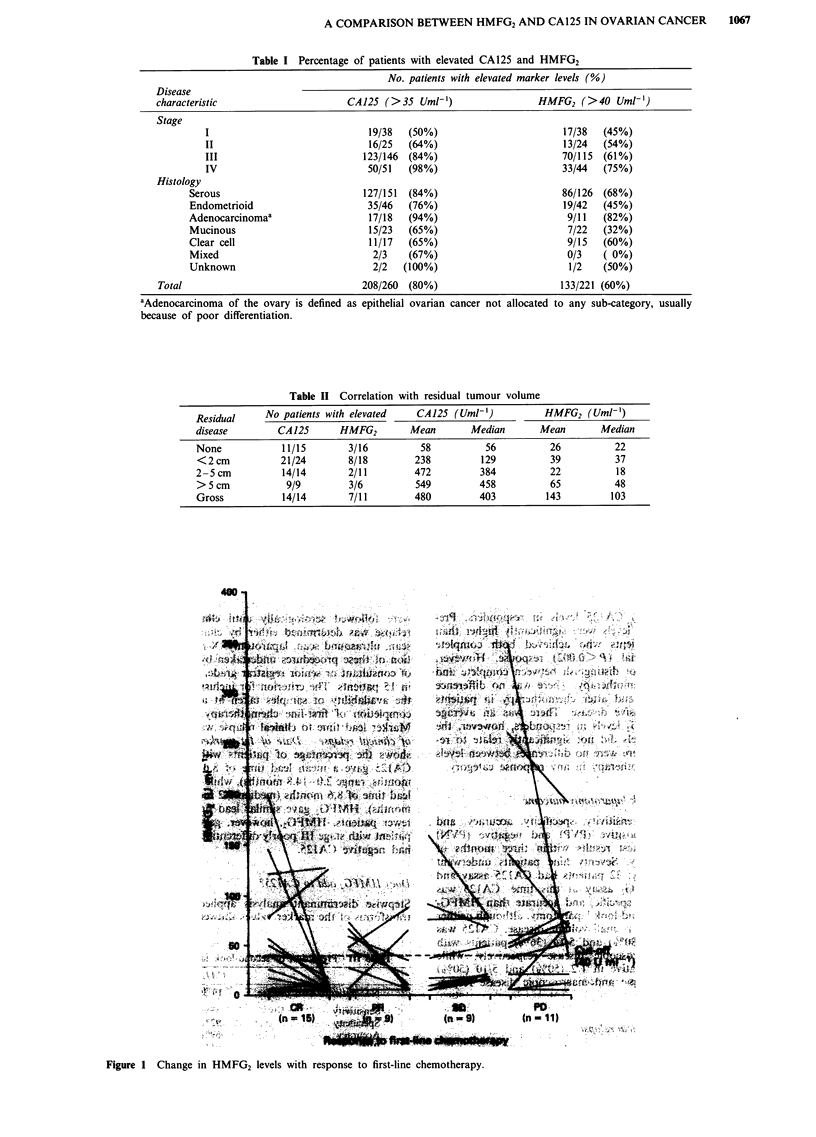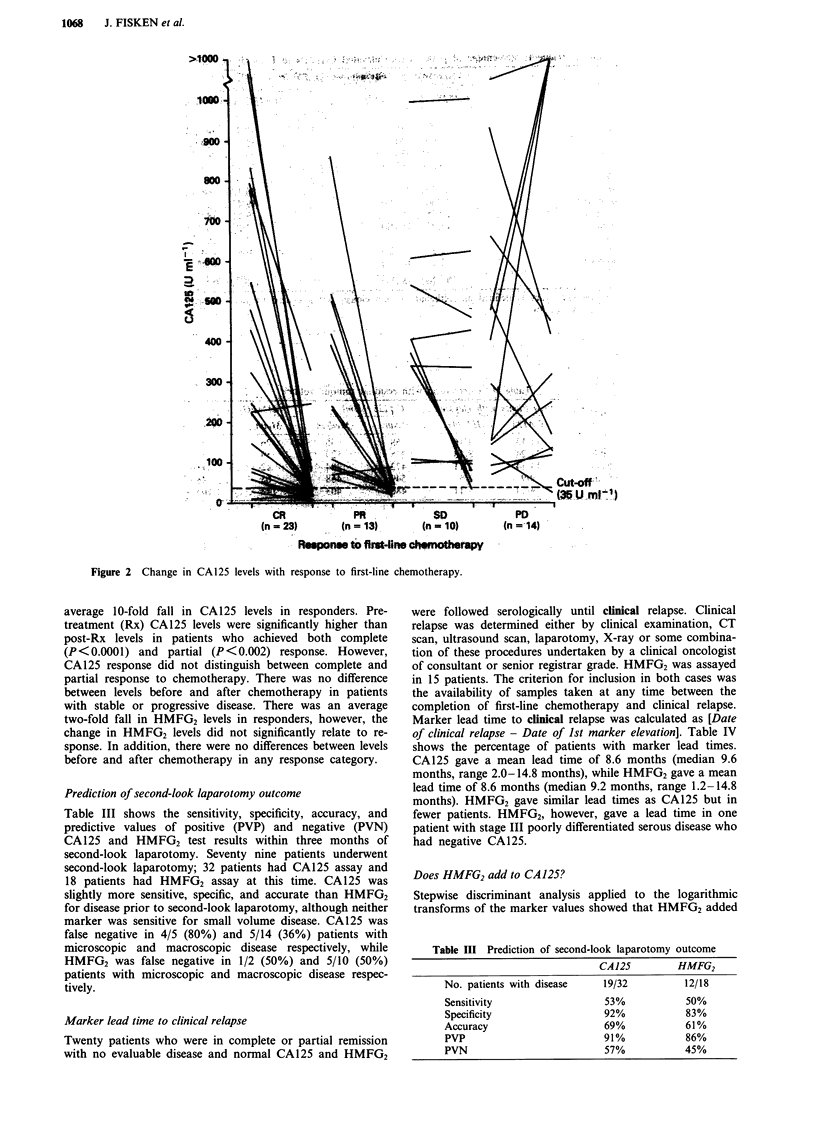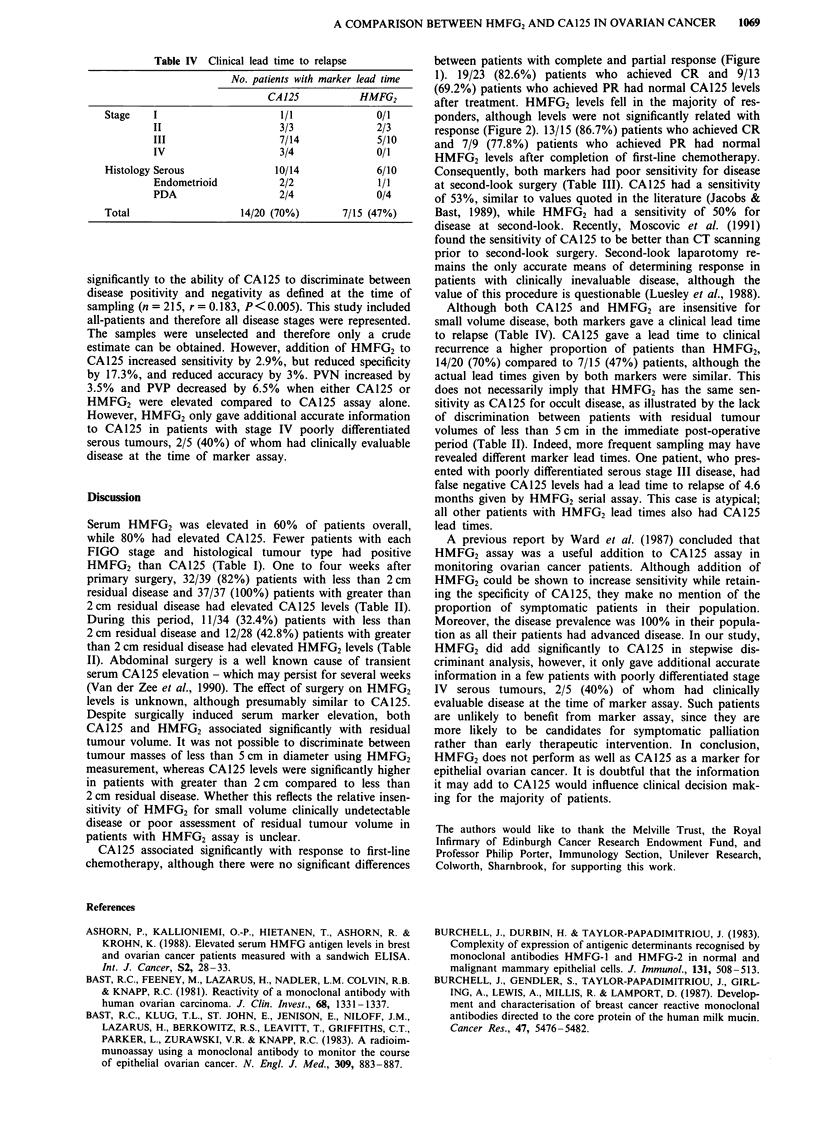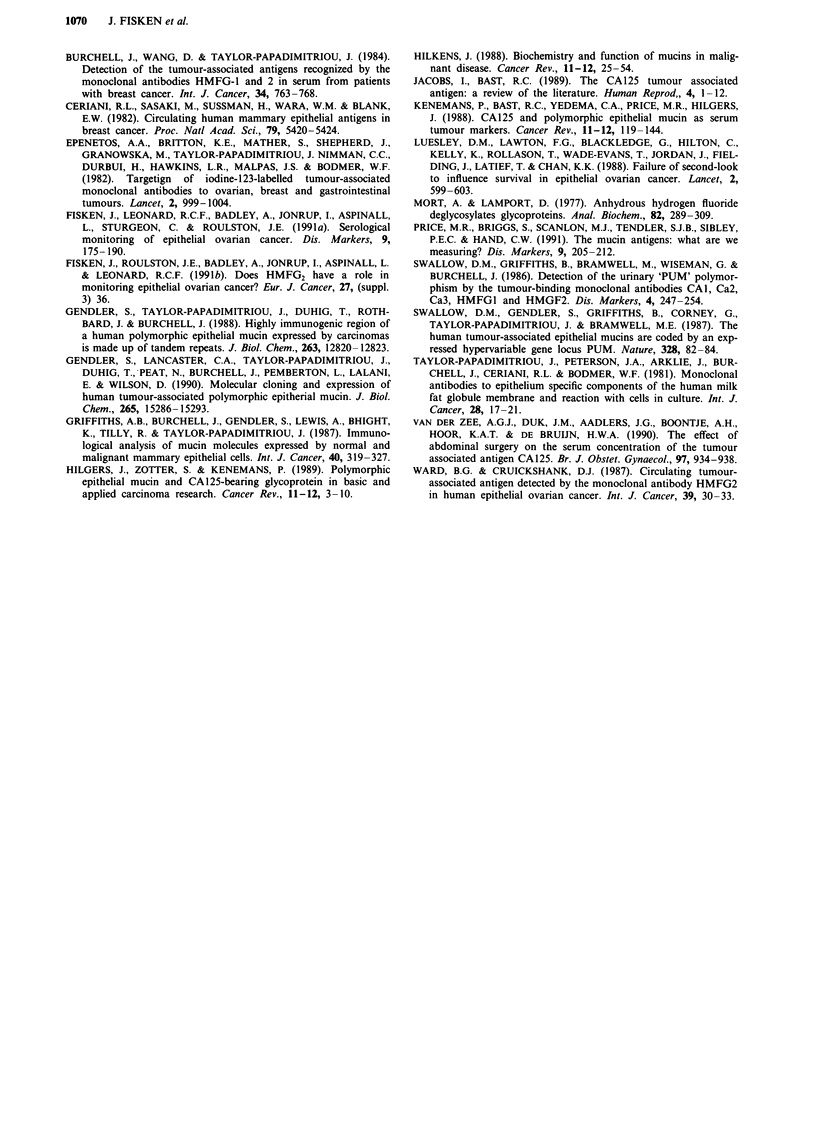# The value of the human milk fat globule membrane antigen HMFG2 in epithelial ovarian cancer monitoring: comparison with CA125.

**DOI:** 10.1038/bjc.1993.195

**Published:** 1993-05

**Authors:** J. Fisken, J. E. Roulston, C. Sturgeon, R. A. Badley, I. Jönrup, L. Aspinall, R. C. Leonard

**Affiliations:** University Department of Clinical Biochemistry, Royal Infirmary, Edinburg, UK.

## Abstract

We assayed serum HMFG2 in serial samples from 215 primary epithelial ovarian cancer patients using an 'in-house' single determinant ELISA, 45% of patients with stage I, 54% with stage II, 61% with stage III and 75% with stage IV disease had elevated serum HMFG2. Post-operative levels were significantly related with residual tumour volume (P < 0.005), and fell in the majority of responders, although the association with response to first-line chemotherapy was not significant. HMFG2 had a sensitivity of 50% specificity of 83%, accuracy of 61%, PVP of 86% and PVN of 45% for disease at second-look laparotomy. Serial levels gave a lead time to clinical relapse in 47% of patients who responded to therapy, including one patient with negative CA125 levels. HMFG, paralleled CA125 in many respects, although it was elevated in fewer patients. In a stepwise discriminant analysis, HMFG2 added to the discrimination of CA125 (r = 0.183, P < 0.005), although additional accurate information was only given in patients with advanced poorly differentiated serous cystadenocarcinoma. Given that HMFG2 is expressed in few patients who are CA125 negative it is unlikely that it will have a significant clinical impact upon patient management.


Br. J. Cancer (1993), 67, 1065–1070 © Macmillan Press Ltd., 1993

### The value of the human milk fat globule membrane antigen $HMFG_2$ in epithelial ovarian cancer monitoring: comparison with CA125

J. Fisken[1,5], J.E. Roulston[1], C. Sturgeon[1], R.A. Badley[3], I. Jönrup[3], L. Aspinall[4] & R.C.F. Leonard[2]

*University Departments of [1]Clinical Biochemistry and [2]Clinical Oncology, Royal Infirmary, Edinburgh, EH3 9YW; [3]Immunology and [4]Information Services Sections, Unilever Research, Colworth, Sharnbrook, Bedford MK44 1LQ, UK.*

**Summary** We assayed serum $HMFG_2$ in serial samples from 215 primary epithelial ovarian cancer patients using an 'in-house' single determinant ELISA. 45% of patients with stage I, 54% with stage II, 61% with stage III and 75% with stage IV disease had elevated serum $HMFG_2$. Post-operative levels were significantly related with residual tumour volume ($P<0.005$), and fell in the majority of responders, although the association with response to first-line chemotherapy was not significant. $HMFG_2$ had a sensitivity of 50% specificity of 83%, accuracy of 61%, PVP of 86% and PVN of 45% for disease at second-look laparotomy. Serial levels gave a lead time to clinical relapse in 47% of patients who responded to therapy, including one patient with negative CA125 levels. $HMFG_2$ paralleled CA125 in many respects, although it was elevated in fewer patients. In a stepwise discriminant analysis, $HMFG_2$ added to the discrimination of CA125 ($r = 0.183$, $P<0.005$), although additional accurate information was only given in patients with advanced poorly differentiated serous cystadenocarcinoma. Given that $HMFG_2$ is expressed in few patients who are CA125 negative it is unlikely that it will have a significant clinical impact upon patient management.

Since its discovery by Bast *et al.* (1981), CA125 has become established as a useful serological marker for monitoring patients with epithelial ovarian cancer. However, approximately 15% of patients do not express CA125 and for the past decade tumour markers that may complement CA125 have been intensively sought (Fisken *et al.*, 1991*a*). The most promising antigenic molecule to date is polymorphic epithelial mucin (PEM), which was originally defined by polyclonal antisera against human milk fat globule preparations by Ceriani *et al.* (1982). Numerous antibodies have been raised against different epitopes found on PEM (Taylor-Papadimitriou *et al.*, 1981, Kenemans *et al.*, 1988, Hilgers *et al.*, 1989, Price *et al.*, 1991).

PEM is a non-gel forming mucin that has been relatively well characterised (for review see Hilkens, 1988). Burchell *et al.* (1983) showed several intermediate-sized glycoproteins in Western blots of cell lysates of T47D breast cancer cells, thought to be long lived intermediates in the glycoslyation process (Griffiths *et al.*, 1987). PEM exhibits extensive polymorphism, first noted at the glycoprotein level (Swallow *et al.*, 1986 and 1987), resulting from genetic polymorphism. Heterogeneity in the size of the apomucin results from a variable number of tandem repeat units in the gene (Gendler *et al.*, 1988) which has been mapped to band 22q of chromosome 1 (Swallow *et al.*, 1987). Recently, Gendler *et al.* (1990) have reported the full sequence of PEM.

The monoclonal antibody to $HMFG_2$ raised by Taylor-Papadimitriou *et al.* (1981) was successfully used initially for *in vivo* imaging of ovarian cancer (Epenetos *et al.*, 1982). Whilst many antibodies are directed to the carbohydrate moiety or a combined carbohydrate-protein epitope, anti-$HMFG_2$ recognises the amino acid sequence asparagine-threonine-arginine (Price *et al.*, 1991). Using this antibody, Burchell *et al.* (1984) originally found elevated serum $HMFG_2$ in 5/6 ovarian cancer patients using an immunoradiometric assay. Two subsequent larger studies found elevated serum $HMFG_2$ in 3/9 (33%) stage I patients and 18/29 (62%) patients with stages II–IV (Ward *et al.*, 1987), and elevated serum HMFG, using antibody designated HMFG III C12, in 15/30 (50%) and 4/34 (12%) patients with and without clinically evident ovarian cancer respectively (Ashorn *et al.*, 1988). In addition, Ward *et al.* (1987) report $HMFG_2$ to be a clinically useful addition to CA125 in patients with advanced ovarian cancer.

In the largest study to date, we have assayed serum samples from 215 epithelial ovarian cancer patients using a single determinant ELISA employing the monoclonal antibody $HMFG_2$ (Fisken *et al.*, 1991*b*). The value of $HMFG_2$ alone in monitoring epithelial ovarian cancer and in addition to CA125 was assessed.

### Materials and methods

### Patients and samples

Blood samples were collected from April 1984 to July 1989 from patients attending the Royal Infirmary and Western General Hospital in Edinburgh. The serum was separated by centrifugation at 1500 *g* for 10 min at room temperature and stored in aliquots at $-20$°C until assay. The patients had a mean age of 59 years at diagnosis (median 59 years, range 23–81 years), and were followed-up on average for 26.7 months (median 20.3 months, range 0.5–173.7 months).

$HMFG_2$ was assayed in 880 serial serum samples from 215 epithelial ovarian cancer patients while CA125 was assayed in 1,237 samples from 250 patients, including those assayed for $HMFG_2$ Table I shows the number of patients with each stage and histological tumour type. The discrepancy in the total number of patients results from assaying patients for CA125 ($n = 10$) and $HMFG_2$ ($n = 6$) during two different disease stages; these patients were counted twice.

### CA125 assay

CA125 was assayed using the CIS IRMA according to manufacturer's instructions. A cut-off value of 35 $U ml^{-1}$ was used, as established by Bast *et al.* (1983).

### $HMFG_2$ assay

(1) *Preparation of $HMFG_2$-horseradish peroxidase conjugate* Horseradish peroxidase (HRP) enzyme was conjugated to $HMFG_2$ IgG in a 1:1 ratio. 5 mg HRP (Sigma Type VI) was dissolved in 1 ml distilled $H_2O$, and oxidised by the addition

Correspondence: J.E. Roulston.
[5]Current address; Cancer Research Campaign Clinical Trials Centre, King's College, London SE5 9NU, UK.
Received 12 May 1992; and in revised form 1 December 1992.

of 0.4 ml freshly prepared 0.1 M sodium metaperiodate for 20 min at room temperature in the dark, while gently stirring occasionally. Oxidised HRP was then dialysed overnight at 4°C with 11 1 mM acetate buffer pH 4.4, stirring continuously. The pH was brought to pH 9.0 with 0.2 M carbonate buffer pH 9.5. 5 mg $HMFG_2$ MAb in 1 ml carbonate buffer pH 9.5 was added and stirred gently for 2 h at room temperature in the dark. 0.1 ml of freshly prepared sodium borohydride (5 mgml$^{-1}$ in distilled $H_2O$) was added and incubated at 4°C for 2 h. The conjugate was finally dialysed with phosphate buffered saline (PBS) pH 7.4 containing 0.01% Thimerosal, and was stored in this buffer at 4°C in the dark.

(2) *Extraction and deglycosylation of $HMFG_2$ for standard preparation*

*(a) Purification of the milk mucin* Human breast milk was centrifuged at 10000 *g* for 30 min to isolate the skimmed milk fraction. HMFG was prepared from human skimmed milk by affinity chromatography on an $HMFG_2$ sepharose column prepared by purification of tissue culture supernatant using a Protein A column and coupling of the purified MAb to cyanogen bromide activated sepharose (Pharmacia) as described by the manufacturer's instructions. Human skimmed milk was passed in batches of 100 ml through the column followed by extensive washing with PBS pH 7.4. Bound antigen was eluted using 0.1 M glycine pH 2.5 and the fractions registering absorbance at 280 nm were pooled and dialysed against 0.25 M acetic acid and freeze dried. Solutions made from this material were stored at − 20°C.

*(b) Deglycosylation of HMFG* Purified HMFG was partially deglycosylated by hydrolysis with anhydrous hydrogen fluoride for 1 h at 4°C, as described by Mort and Lamport (1977). Longer incubation results in complete deglycosylation of HMFG which does not react as strongly with $HMFG_2$ antibody.

*(c) Preparation of $HMFG_2$ standards* $HMFG_2$ standards were prepared by the method of Burchell *et al.* (1987) in PBS pH 7.4, containing 7% bovine serum albumin (BSA) and 0.01% merthiolate as preservative, to avoid interference from HMFG normally present at varying levels in normal human sera. HMFG concentrations were set by reference to an original preparation isolated by Dr S. Mather (St Bartholomew's Hospital, London). One mg of the freeze dried powder was arbitrarily equal to $10^6$ units. Deglycosylated HMFG was calibrated against a preparation obtained from Dr J. Taylor-Papadimitriou (ICRF, Lincoln's Inn Fields, London) in a similar way. Aliquoted standards were stable for at least 2 weeks at 4°C, whilst deglycosylated HMFG kept for only 1 week at 4°C.

(3) *$HMFG_2$ assay protocol and quality assessment*
All samples were assayed in duplicate. Microtitre plates (M129B, Dynatech, Billingshurst, Kent, UK) were coated overnight at 4°C with 50 µl 5.0 µgml$^{-1}$ $HMFG_2$ MAb in 0.05 M carbonate buffer pH 9.6. The plates were washed three times with 100 µl 0.15 M PBS pH 7.4 containing 0.05% polyoxyethylene sorbitan monolaurate (Tween 20) (PBS/Tween 20). 25 µl neat serum, standard, or control were incubated with 25 µl PBS/Tween 20 for 30 min at 37°C in a shaking incubator (Dynatech). The plates were washed three times with PBS/Tween 20, and 50 µl $HMFG_2$ MAb-HRP conjugate (see 4.7.3) at 1:1000 in PBS/Tween 20 incubated for 30 min at 37°C. After three final washes, 100 µl peroxidase substrate: 0.04% w/v O-phenylene-diamine and 0.02% v/v $H_2O_2$ in 0.15 M citrate phosphate buffer pH 5.0 was added. The reaction was stopped after 30 min incubation at 37°C with the addition of 50 µl 2.5 M $H_2SO_4$, and optical density determined at 492 nm with a Titertek Multiscan (Fisken *et al.*, 1991*b*).

Control and unknown sample concentrations were interpolated manually from dose-response curves constructed using standard $HMFG_2$ preparations at the following concentrations: 0, 50, 100, 200, 350, and 500 Uml$^{-1}$. The coefficients of variation were calculated using two controls (50 Uml$^{-1}$ and 150 Uml$^{-1}$). The inter-assay CVs ($n = 22$) for low and high concentrations were 6.8% and 4.9% respectively, while the intra-assay CVs ($n = 22$) for the low and high controls were 14.9% and 6.6% respectively. A cut-off value of 40 Uml$^{-1}$ was chosen empirically to give the best discrimination between patients with active disease and those with no disease activity. Five per cent (6/121) of apparently healthy blood donors had levels greater than this value.

*Statistics*

A database containing all the patients case histories and serial CA125 and $HMFG_2$ levels was developed using computing facilities at Unilever Research, Colworth Laboratories. The F test (one way analysis of variance) was used to determine the association between post-operative residual disease and marker levels after surgery. The Kruskal Wallis test was used to determine the relationship with overall response to chemotherapy, and the difference between complete and partial responders determined by the Mann Whitney test. The Wilcoxon Signed Rank test was used to determine the difference between marker levels before and after first-line treatment.

All parametric tests were based upon logarithmic transforms of marker values. The median values quoted are back-transforms of the mean logarithms. Non-parametric analyses were carried out in parallel but, as they were in agreement with the parametric analyses in all cases, the latter were preferred as being more informative.

Tests on marker change were also applied to logarithmic transforms and therefore correspond to absolute differences on this scale but relative changes on the original linear scale.

### Results

*Percentage of patients with raised CA125 and $HMFG_2$*

The physio-pathological cut-offs for CA125 and $HMFG_2$ were based upon the level of analyte below which 95% of disease-free controls fell (Bast *et al.*, 1983; Fisken *et al.*, 1991*a*). In both cases effectiveness was confirmed with Receiver Operating Characteristic (ROC) plots. The proportion of patients with elevated CA125 and $HMFG_2$ increased with advancing stage, and a higher proportion of patients with serous tumours and adenocarcinomas had elevated CA125 and $HMFG_2$, see (Table I). CA125 was however elevated in a higher proportion of patients with all disease stages and tumour types than $HMFG_2$.

*Marker relationship with tumour burden*

Table II shows mean and median levels of CA125 and $HMFG_2$ and the proportion of patients with elevated levels 1–4 weeks after primary laparotomy (mean 17.8 days, median 18 days, range 3–38 days) in patients with different amounts of residual disease. Both CA125 and $HMFG_2$ showed a significant association with tumour burden ($P < 0.0001$ and $P < 0.005$ respectively). Patients with residual disease > 2 cm had significantly higher CA125 levels that those with < 2 cm residual disease ($P < 0.0001$). $HMFG_2$ was less sensitive, patients with > 5 cm had significantly higher $HMFG_2$ levels than those with < 5 cm ($P < 0.005$). $HMFG_2$ was unable to discriminate between tumour burdens < 5 cm.

*Marker relationship with response to primary chemotherapy*

Figures 1 and 2 show the changes in CA125 and $HMFG_2$ levels respectively from pretreatment to post-treatment in patients who received first-line chemotherapy (irrespective of drug regime). CA125 showed a significant association with overall response to chemotherapy ($P < 0.005$). There was an

**Table I** Percentage of patients with elevated CA125 and $HMFG_2$

| *Disease characteristic* | *No. patients with elevated marker levels (%)* | |
|---|---|---|
| | *CA125 (>35 Uml⁻¹)* | *$HMFG_2$ (>40 Uml⁻¹)* |
| *Stage* | | |
| I | 19/38 (50%) | 17/38 (45%) |
| II | 16/25 (64%) | 13/24 (54%) |
| III | 123/146 (84%) | 70/115 (61%) |
| IV | 50/51 (98%) | 33/44 (75%) |
| *Histology* | | |
| Serous | 127/151 (84%) | 86/126 (68%) |
| Endometrioid | 35/46 (76%) | 19/42 (45%) |
| Adenocarcinoma[a] | 17/18 (94%) | 9/11 (82%) |
| Mucinous | 15/23 (65%) | 7/22 (32%) |
| Clear cell | 11/17 (65%) | 9/15 (60%) |
| Mixed | 2/3 (67%) | 0/3 ( 0%) |
| Unknown | 2/2 (100%) | 1/2 (50%) |
| *Total* | 208/260 (80%) | 133/221 (60%) |

[a]Adenocarcinoma of the ovary is defined as epithelial ovarian cancer not allocated to any sub-category, usually because of poor differentiation.

**Table II** Correlation with residual tumour volume

| *Residual disease* | *No patients with elevated* | | *CA125 (Uml⁻¹)* | | *$HMFG_2$ (Uml⁻¹)* | |
|---|---|---|---|---|---|---|
| | *CA125* | *$HMFG_2$* | *Mean* | *Median* | *Mean* | *Median* |
| None | 11/15 | 3/16 | 58 | 56 | 26 | 22 |
| <2 cm | 21/24 | 8/18 | 238 | 129 | 39 | 37 |
| 2–5 cm | 14/14 | 2/11 | 472 | 384 | 22 | 18 |
| >5 cm | 9/9 | 3/6 | 549 | 458 | 65 | 48 |
| Gross | 14/14 | 7/11 | 480 | 403 | 143 | 103 |

**Figure 1** Change in $HMFG_2$ levels with response to first-line chemotherapy.

**Figure 2** Change in CA125 levels with response to first-line chemotherapy.

average 10-fold fall in CA125 levels in responders. Pre-treatment (Rx) CA125 levels were significantly higher than post-Rx levels in patients who achieved both complete ($P<0.0001$) and partial ($P<0.002$) response. However, CA125 response did not distinguish between complete and partial response to chemotherapy. There was no difference between levels before and after chemotherapy in patients with stable or progressive disease. There was an average two-fold fall in $HMFG_2$ levels in responders, however, the change in $HMFG_2$ levels did not significantly relate to response. In addition, there were no differences between levels before and after chemotherapy in any response category.

*Prediction of second-look laparotomy outcome*

Table III shows the sensitivity, specificity, accuracy, and predictive values of positive (PVP) and negative (PVN) CA125 and $HMFG_2$ test results within three months of second-look laparotomy. Seventy nine patients underwent second-look laparotomy; 32 patients had CA125 assay and 18 patients had $HMFG_2$ assay at this time. CA125 was slightly more sensitive, specific, and accurate than $HMFG_2$ for disease prior to second-look laparotomy, although neither marker was sensitive for small volume disease. CA125 was false negative in 4/5 (80%) and 5/14 (36%) patients with microscopic and macroscopic disease respectively, while $HMFG_2$ was false negative in 1/2 (50%) and 5/10 (50%) patients with microscopic and macroscopic disease respectively.

*Marker lead time to clinical relapse*

Twenty patients who were in complete or partial remission with no evaluable disease and normal CA125 and $HMFG_2$ were followed serologically until **clinical** relapse. Clinical relapse was determined either by clinical examination, CT scan, ultrasound scan, laparotomy, X-ray or some combination of these procedures undertaken by a clinical oncologist of consultant or senior registrar grade. $HMFG_2$ was assayed in 15 patients. The criterion for inclusion in both cases was the availability of samples taken at any time between the completion of first-line chemotherapy and clinical relapse. Marker lead time to **clinical** relapse was calculated as [*Date of clinical relapse – Date of 1st marker elevation*]. Table IV shows the percentage of patients with marker lead times. CA125 gave a mean lead time of 8.6 months (median 9.6 months, range 2.0–14.8 months), while $HMFG_2$ gave a mean lead time of 8.6 months (median 9.2 months, range 1.2–14.8 months). $HMFG_2$ gave similar lead times as CA125 but in fewer patients. $HMFG_2$, however, gave a lead time in one patient with stage III poorly differentiated serous disease who had negative CA125.

*Does $HMFG_2$ add to CA125?*

Stepwise discriminant analysis applied to the logarithmic transforms of the marker values showed that $HMFG_2$ added

**Table III** Prediction of second-look laparotomy outcome

| | *CA125* | *$HMFG_2$* |
|---|---|---|
| No. patients with disease | 19/32 | 12/18 |
| Sensitivity | 53% | 50% |
| Specificity | 92% | 83% |
| Accuracy | 69% | 61% |
| PVP | 91% | 86% |
| PVN | 57% | 45% |

**Table IV** Clinical lead time to relapse

| | | No. patients with marker lead time | |
|---|---|---|---|
| | | *CA125* | *$HMFG_2$* |
| Stage | I | 1/1 | 0/1 |
| | II | 3/3 | 2/3 |
| | III | 7/14 | 5/10 |
| | IV | 3/4 | 0/1 |
| Histology | Serous | 10/14 | 6/10 |
| | Endometrioid | 2/2 | 1/1 |
| | PDA | 2/4 | 0/4 |
| Total | | 14/20 (70%) | 7/15 (47%) |

significantly to the ability of CA125 to discriminate between disease positivity and negativity as defined at the time of sampling ($n = 215$, $r = 0.183$, $P < 0.005$). This study included all-patients and therefore all disease stages were represented. The samples were unselected and therefore only a crude estimate can be obtained. However, addition of $HMFG_2$ to CA125 increased sensitivity by 2.9%, but reduced specificity by 17.3%, and reduced accuracy by 3%. PVN increased by 3.5% and PVP decreased by 6.5% when either CA125 or $HMFG_2$ were elevated compared to CA125 assay alone. However, $HMFG_2$ only gave additional accurate information to CA125 in patients with stage IV poorly differentiated serous tumours, 2/5 (40%) of whom had clinically evaluable disease at the time of marker assay.

### Discussion

Serum $HMFG_2$ was elevated in 60% of patients overall, while 80% had elevated CA125. Fewer patients with each FIGO stage and histological tumour type had positive $HMFG_2$ than CA125 (Table I). One to four weeks after primary surgery, 32/39 (82%) patients with less than 2 cm residual disease and 37/37 (100%) patients with greater than 2 cm residual disease had elevated CA125 levels (Table II). During this period, 11/34 (32.4%) patients with less than 2 cm residual disease and 12/28 (42.8%) patients with greater than 2 cm residual disease had elevated $HMFG_2$ levels (Table II). Abdominal surgery is a well known cause of transient serum CA125 elevation – which may persist for several weeks (Van der Zee *et al*., 1990). The effect of surgery on $HMFG_2$ levels is unknown, although presumably similar to CA125. Despite surgically induced serum marker elevation, both CA125 and $HMFG_2$ associated significantly with residual tumour volume. It was not possible to discriminate between tumour masses of less than 5 cm in diameter using $HMFG_2$ measurement, whereas CA125 levels were significantly higher in patients with greater than 2 cm compared to less than 2 cm residual disease. Whether this reflects the relative insensitivity of $HMFG_2$ for small volume clinically undetectable disease or poor assessment of residual tumour volume in patients with $HMFG_2$ assay is unclear.

CA125 associated significantly with response to first-line chemotherapy, although there were no significant differences between patients with complete and partial response (Figure 1). 19/23 (82.6%) patients who achieved CR and 9/13 (69.2%) patients who achieved PR had normal CA125 levels after treatment. $HMFG_2$ levels fell in the majority of responders, although levels were not significantly related with response (Figure 2). 13/15 (86.7%) patients who achieved CR and 7/9 (77.8%) patients who achieved PR had normal $HMFG_2$ levels after completion of first-line chemotherapy. Consequently, both markers had poor sensitivity for disease at second-look surgery (Table III). CA125 had a sensitivity of 53%, similar to values quoted in the literature (Jacobs & Bast, 1989), while $HMFG_2$ had a sensitivity of 50% for disease at second-look. Recently, Moscovic *et al*. (1991) found the sensitivity of CA125 to be better than CT scanning prior to second-look surgery. Second-look laparotomy remains the only accurate means of determining response in patients with clinically inevaluable disease, although the value of this procedure is questionable (Luesley *et al*., 1988).

Although both CA125 and $HMFG_2$ are insensitive for small volume disease, both markers gave a clinical lead time to relapse (Table IV). CA125 gave a lead time to clinical recurrence a higher proportion of patients than $HMFG_2$, 14/20 (70%) compared to 7/15 (47%) patients, although the actual lead times given by both markers were similar. This does not necessarily imply that $HMFG_2$ has the same sensitivity as CA125 for occult disease, as illustrated by the lack of discrimination between patients with residual tumour volumes of less than 5 cm in the immediate post-operative period (Table II). Indeed, more frequent sampling may have revealed different marker lead times. One patient, who presented with poorly differentiated serous stage III disease, had false negative CA125 levels had a lead time to relapse of 4.6 months given by $HMFG_2$ serial assay. This case is atypical; all other patients with $HMFG_2$ lead times also had CA125 lead times.

A previous report by Ward *et al*. (1987) concluded that $HMFG_2$ assay was a useful addition to CA125 assay in monitoring ovarian cancer patients. Although addition of $HMFG_2$ could be shown to increase sensitivity while retaining the specificity of CA125, they make no mention of the proportion of symptomatic patients in their population. Moreover, the disease prevalence was 100% in their population as all their patients had advanced disease. In our study, $HMFG_2$ did add significantly to CA125 in stepwise discriminant analysis, however, it only gave additional accurate information in a few patients with poorly differentiated stage IV serous tumours, 2/5 (40%) of whom had clinically evaluable disease at the time of marker assay. Such patients are unlikely to benefit from marker assay, since they are more likely to be candidates for symptomatic palliation rather than early therapeutic intervention. In conclusion, $HMFG_2$ does not perform as well as CA125 as a marker for epithelial ovarian cancer. It is doubtful that the information it may add to CA125 would influence clinical decision making for the majority of patients.

The authors would like to thank the Melville Trust, the Royal Infirmary of Edinburgh Cancer Research Endowment Fund, and Professor Philip Porter, Immunology Section, Unilever Research, Colworth, Sharnbrook, for supporting this work.

### References

ASHORN, P., KALLIONIEMI, O.-P., HIETANEN, T., ASHORN, R. & KROHN, K. (1988). Elevated serum HMFG antigen levels in brest and ovarian cancer patients measured with a sandwich ELISA. *Int. J. Cancer*, **S2**, 28–33.

BAST, R.C., FEENEY, M., LAZARUS, H., NADLER, L.M. COLVIN, R.B. & KNAPP, R.C. (1981). Reactivity of a monoclonal antibody with human ovarian carcinoma. *J. Clin. Invest.*, **68**, 1331–1337.

BAST, R.C., KLUG, T.L., ST. JOHN, E., JENISON, E., NILOFF, J.M., LAZARUS, H., BERKOWITZ, R.S., LEAVITT, T., GRIFFITHS, C.T., PARKER, L., ZURAWSKI, V.R. & KNAPP, R.C. (1983). A radioimmunoassay using a monoclonal antibody to monitor the course of epithelial ovarian cancer. *N. Engl. J. Med.*, **309**, 883–887.

BURCHELL, J., DURBIN, H. & TAYLOR-PAPADIMITRIOU, J. (1983). Complexity of expression of antigenic determinants recognised by monoclonal antibodies HMFG-1 and HMFG-2 in normal and malignant mammary epithelial cells. *J. Immunol.*, **131**, 508–513.

BURCHELL, J., GENDLER, S., TAYLOR-PAPADIMITRIOU, J., GIRLING, A., LEWIS, A., MILLIS, R. & LAMPORT, D. (1987). Development and characterisation of breast cancer reactive monoclonal antibodies directed to the core protein of the human milk mucin. *Cancer Res.*, **47**, 5476–5482.

BURCHELL, J., WANG, D. & TAYLOR-PAPADIMITRIOU, J. (1984). Detection of the tumour-associated antigens recognized by the monoclonal antibodies HMFG-1 and 2 in serum from patients with breast cancer. *Int. J. Cancer*, **34**, 763–768.

CERIANI, R.L., SASAKI, M., SUSSMAN, H., WARA, W.M. & BLANK, E.W. (1982). Circulating human mammary epithelial antigens in breast cancer. *Proc. Natl Acad. Sci.*, **79**, 5420–5424.

EPENETOS, A.A., BRITTON, K.E., MATHER, S., SHEPHERD, J., GRANOWSKA, M., TAYLOR-PAPADIMITRIOU, J. NIMMAN, C.C., DURBUI, H., HAWKINS, L.R., MALPAS, J.S. & BODMER, W.F. (1982). Targetign of iodine-123-labelled tumour-associated monoclonal antibodies to ovarian, breast and gastrointestinal tumours. *Lancet*, **2**, 999–1004.

FISKEN, J., LEONARD, R.C.F., BADLEY, A., JONRUP, I., ASPINALL, L., STURGEON, C. & ROULSTON, J.E. (1991*a*). Serological monitoring of epithelial ovarian cancer. *Dis. Markers*, **9**, 175–190.

FISKEN, J., ROULSTON, J.E., BADLEY, A., JONRUP, I., ASPINALL, L. & LEONARD, R.C.F. (1991*b*). Does $HMFG_2$ have a role in monitoring epithelial ovarian cancer? *Eur. J. Cancer*, **27**, (suppl. 3) 36.

GENDLER, S., TAYLOR-PAPADIMITRIOU, J., DUHIG, T., ROTHBARD, J. & BURCHELL, J. (1988). Highly immunogenic region of a human polymorphic epithelial mucin expressed by carcinomas is made up of tandem repeats. *J. Biol. Chem.*, **263**, 12820–12823.

GENDLER, S., LANCASTER, C.A., TAYLOR-PAPADIMITRIOU, J., DUHIG, T., PEAT, N., BURCHELL, J., PEMBERTON, L., LALANI, E. & WILSON, D. (1990). Molecular cloning and expression of human tumour-associated polymorphic epitherial mucin. *J. Biol. Chem.*, **265**, 15286–15293.

GRIFFITHS, A.B., BURCHELL, J., GENDLER, S., LEWIS, A., BHIGHT, K., TILLY, R. & TAYLOR-PAPADIMITRIOU, J. (1987). Immunological analysis of mucin molecules expressed by normal and malignant mammary epithelial cells. *Int. J. Cancer*, **40**, 319–327.

HILGERS, J., ZOTTER, S. & KENEMANS, P. (1989). Polymorphic epithelial mucin and CA125-bearing glycoprotein in basic and applied carcinoma research. *Cancer Rev.*, **11–12**, 3–10.

HILKENS, J. (1988). Biochemistry and function of mucins in malignant disease. *Cancer Rev.*, **11–12**, 25–54.

JACOBS, I., BAST, R.C. (1989). The CA125 tumour associated antigen: a review of the literature. *Human Reprod,*, **4**, 1–12.

KENEMANS, P., BAST, R.C., YEDEMA, C.A., PRICE, M.R., HILGERS, J. (1988). CA125 and polymorphic epithelial mucin as serum tumour markers. *Cancer Rev.*, **11–12**, 119–144.

LUESLEY, D.M., LAWTON, F.G., BLACKLEDGE, G., HILTON, C., KELLY, K., ROLLASON, T., WADE-EVANS, T., JORDAN, J., FIELDING, J., LATIEF, T. & CHAN, K.K. (1988). Failure of second-look to influence survival in epithelial ovarian cancer. *Lancet*, **2**, 599–603.

MORT, A. & LAMPORT, D. (1977). Anhydrous hydrogen fluoride deglycosylates glycoproteins. *Anal. Biochem.*, **82**, 289–309.

PRICE, M.R., BRIGGS, S., SCANLON, M.J., TENDLER, S.J.B., SIBLEY, P.E.C. & HAND, C.W. (1991). The mucin antigens: what are we measuring? *Dis. Markers*, **9**, 205–212.

SWALLOW, D.M., GRIFFITHS, B., BRAMWELL, M., WISEMAN, G. & BURCHELL, J. (1986). Detection of the urinary 'PUM' polymorphism by the tumour-binding monoclonal antibodies CA1, Ca2, Ca3, HMFG1 and HMGF2. *Dis. Markers*, **4**, 247–254.

SWALLOW, D.M., GENDLER, S., GRIFFITHS, B., CORNEY, G., TAYLOR-PAPADIMITRIOU, J. & BRAMWELL, M.E. (1987). The human tumour-associated epithelial mucins are coded by an expressed hypervariable gene locus PUM. *Nature*, **328**, 82–84.

TAYLOR-PAPADIMITRIOU, J., PETERSON, J.A., ARKLIE, J., BURCHELL, J., CERIANI, R.L. & BODMER, W.F. (1981). Monoclonal antibodies to epithelium specific components of the human milk fat globule membrane and reaction with cells in culture. *Int. J. Cancer*, **28**, 17–21.

VAN DER ZEE, A.G.J., DUK, J.M., AADLERS, J.G., BOONTJE, A.H., HOOR, K.A.T. & DE BRUIJN, H.W.A. (1990). The effect of abdominal surgery on the serum concentration of the tumour associated antigen CA125. *Br. J. Obstet. Gynaecol.*, **97**, 934–938.

WARD, B.G. & CRUICKSHANK, D.J. (1987). Circulating tumour-associated antigen detected by the monoclonal antibody HMFG2 in human epithelial ovarian cancer. *Int. J. Cancer*, **39**, 30–33.